# NSIT: Novel Sequence Identification Tool

**DOI:** 10.1371/journal.pone.0108011

**Published:** 2014-09-29

**Authors:** Benjarath Pupacdi, Asif Javed, Mohammed J. Zaki, Mathuros Ruchirawat

**Affiliations:** 1 Translational Research Unit, Chulabhorn Research Institute, Bangkok, Thailand; 2 Computational and Systems Biology Group, Genome Institute of Singapore, Agency for Science, Technology and Research, Singapore, Singapore; 3 Computer Science Department, Rensselaer Polytechnic Institute, Troy, New York, United States of America; 4 Qatar Computing Research Institute, Doha, Qatar; Univeristy of California Riverside, United States of America

## Abstract

Novel sequences are DNA sequences present in an individual's genome but absent in the human reference assembly. They are predicted to be biologically important, both individual and population specific, and consistent with the known human migration paths. Recent works have shown that an average person harbors 2–5 Mb of such sequences and estimated that the human pan-genome contains as high as 19–40 Mb of novel sequences. To identify them in a *de novo* genome assembly, some existing sequence aligners have been used but no computational method has been specifically proposed for this task. In this work, we developed **NSIT** (**N**ovel **S**equence **I**dentification **T**ool), a software that can accurately and efficiently identify novel sequences in an individual's *de novo* whole genome assembly. We identified and characterized 1.1 Mb, 1.2 Mb, and 1.0 Mb of novel sequences in NA18507 (African), YH (Asian), and NA12878 (European) *de novo* genome assemblies, respectively. Our results show very high concordance with the previous work using the respective reference assembly. In addition, our results using the latest human reference assembly suggest that the amount of novel sequences per individual may not be as high as previously reported. We additionally developed a graphical viewer for comparisons of novel sequence contents. The viewer also helped in identifying sequence contamination; we found 130 kb of Epstein-Barr virus sequence in the previously published NA18507 novel sequences as well as 287 kb of zebrafish repeats in NA12878 *de novo* assembly. NSIT requires 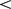
2GB of RAM and 1.5–2 hrs on a commodity desktop. The program is applicable to input assemblies with varying contig/scaffold sizes, ranging from 100 bp to as high as 50 Mb. It works in both 32-bit and 64-bit systems and outperforms, by large margins, other fast sequence aligners previously applied to this task. To our knowledge, NSIT is the first software designed specifically for novel sequence identification in a *de novo* human genome assembly.

## Introduction

Novel sequences are DNA sequences present in at least one human individual but absent in the reference genome. Typically, they are defined as sequences that are 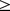
100 bp long, with <90% sequence identity to the reference genome, and not known repeats as their paralogs would exist elsewhere in the reference sequence. The novel sequences are predicted to be functionally important, individual and population specific, and consistent with the known human migration paths [1]. Recent works have identified 2.4–5.1 Mb of novel sequences in the following 3 genomes: YH (Chinese male) [1], NA18507 (Yoruba male) [1], [2], and NA18943 (Japanese male) [3]. It is estimated that the human pan-genome contains 19–40 Mb of novel sequences [1]. These discoveries suggest that a considerable amount of large genetic variation in the human genome remains undiscovered. Hence, there is a need for methods that accurately and efficiently detect novel sequences in a human genome to facilitate comprehensive genome analysis.

To date, three main approaches have been adopted. In the first approach, the *de novo* assembly of a human genome is aligned to the reference sequence and the final unaligned regions that are not masked as known repeats are considered novel. Due to the large size of the human genome, this process usually requires a pipeline of aligners; the most rapid but least sensitive one is applied first to search for regions likely to be homologous, and then more detailed alignments follow via subsequent aligners on the previously defined homologous regions as well as the unaligned regions. The novel sequences of NA18507 (4.8 Mb) and YH (5.1 Mb) were identified via this approach [1] using BLAT, LASTZ, and BLASTn aligners [4]–[6]. In the second approach, first the reference-based whole genome assembly of an individual is constructed, and then *de novo* assembly is carried out on the unmapped reads to create novel sequences. Around 3 Mb of NA18943 novel sequences were discovered with this approach [3] using ABySS, SOAPdenovo, and Velvet assemblers [7]–[9]. Note that constructing the assembly is not a prerequisite to novel sequence search. The last approach targets paired-end read data from next-generation sequencing (NGS) platforms. NovelSeq [2] is currently the only software in this category. The algorithm filters out the reads mappable to the reference sequence using a short-read aligner. *De novo* assembly is performed on the unmapped reads to generate novel sequences, which are then anchored to the reference genome to determine their positions. NovelSeq uses EULER-SR [10] and ABySS assemblers and discovered 2.4 Mb and 2.7 Mb of novel sequences, respectively, in the genome of NA18507. In all three approaches, known repeats can be removed from candidate novel sequences by programs such as RepeatMasker [11].

These approaches offer different algorithmic flavors in finding novel sequences. The first route takes a *de novo* whole genome assembly as an input, and therefore is less reliant on the sequencing technology. Genomic locations of novel sequences are determined as a result of the alignment. The second and third approaches map sequence reads to the reference sequence and acquire those unmapped as input. Genomic positions of novel sequences are also determined in the third approach, but not the second. Novel sequences are difficult to identify precisely due to the complexity of the human genome. The results depend on various factors such as the choice of sequencing technology, programs in the pipelines, and the reference assembly used. For example, the above approaches give differing albeit much overlapping results for the same assembly NA18507, i.e., 4.8 Mb, 2.4 Mb, and 2.7 Mb [1], [2], despite comparing against the same reference sequence. A combination of approaches should thus be used for a more comprehensive evaluation.

Our contribution here is in the first category. NSIT is a fast and accurate aligner with the primary focus of identifying novel sequences in a *de novo* human genome assembly. To our knowledge, it is the first algorithm that specifically targets this problem. NSIT runs in both 32-bit and 64-bit environments, requires <2GB of RAM. Combining NSIT with BLASTn and RepeatMasker, we identified 1.1 Mb, 1.2 Mb, and 1.0 Mb of novel sequences in the *de novo* genome assemblies of NA18507, YH, and NA12878, respectively. NSIT took 1.5–2 hours on a commodity desktop, and is faster than BLAT and LASTZ by large margins. Our results exhibit very high overlap with the respective published results. In addition, when aligning against the latest reference assembly, they suggest that the amount of novel sequences in an individual's genome may not be as high as previously reported. Included with NSIT is a novel sequence graphical viewer to aid in studying the overlap of novel sequences with other set of selected sequences. In addition, the viewer allows for quick visual inspection of sequence contamination; we unveiled 130 kb of Epstein-Barr virus sequences in the NA18507 novel sequences earlier reported in [1] and 287 kb of Zebrafish repeats in NA12878 *de novo* genome assembly. With the rapid growth of personalized genomics and NGS technology, NSIT can be effectively incorporated into existing pipelines for variant detection in human genomes to aid further comprehensive analysis.

## Design and Implementation

NSIT consists of 3 phases: 1) *k*-mer hash table construction, 2) chromosome assignment, and 3) query alignment. Details are discussed below.

### *K*-Mer Hash Table Construction

Any two human genomes share 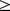
99% of the DNA sequence. Aligning such highly homologous sequences generally uses *k*-mer matches as alignment seeds. NSIT begins by constructing *k*-mer hash tables to index the reference sequence. Each table maps *all* possible *k*-mers in the chromosome string to their corresponding positions in the string.

Let 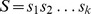 be a *k*-mer, 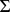 denotes the DNA alphabet set 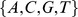, and 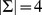. Define 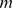 be a 1-to-1 function from 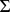 to 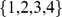 such that 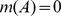, 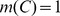, 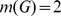, and 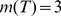. The hash function is defined as 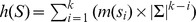. To populate a table 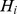 for a reference chromosome 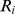, 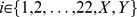, the algorithm scans 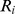 from left to right one base at a time and incrementally adds the current position into the hash table based on the hash value of the *k*-mer starting at that position. The value of *k* is usually set to be between 8–16 but more often a multiple of 3, which is the codon size. For a reasonable trade-off between memory requirements and time, we experimented with *k* values from 10 to 12 and found that the largest hash table, which was that of the human chromosome 1, required about 1 GB of on-disk storage space. The memory usage during the table construction was about 1.5 GB. The hash tables are built and stored on disk one chromosome at a time. The one-time cost for constructing them, as well as their disk-space requirement, is linear to the reference genome size.

### Chromosome Assignment

A human genome *de novo* assembly typically comprises of anywhere between a few thousand to several hundred thousand contigs and scaffolds. The sizes of these sequences can vary considerably and their positions with respect to the reference genome are not known. The chromosome assignment phase aims to identify the candidate target chromosomes and orientations for these contigs and scaffolds, in order to facilitate the finer alignments in the next phase.

**Figure 1:**
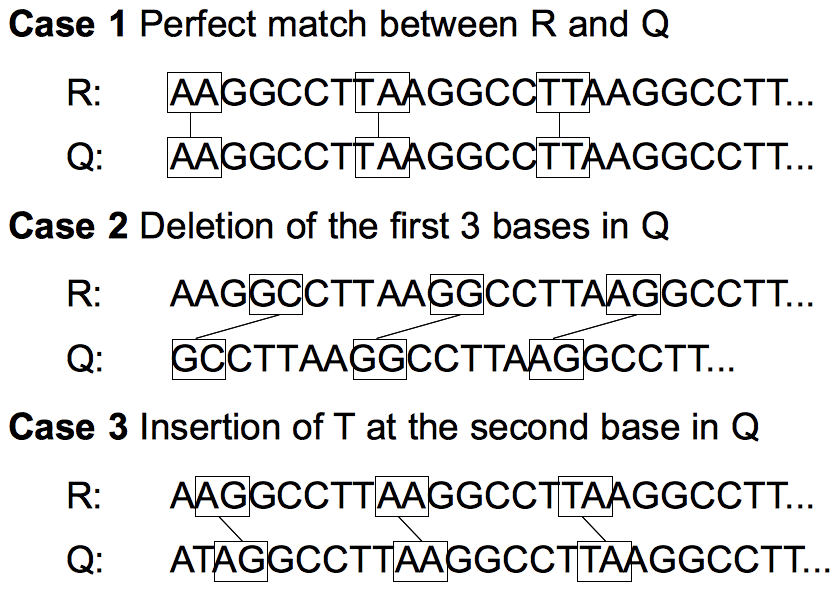
Using non-consecutive *k*-mers to align a *de novo* query sequence Q to a reference sequence R. Case 1 illustrates a perfect match. Cases 2 and 3 show a deletion and an insertion in the query sequence, respectively. Each case requires a different subset of *k*-mers.

Due to the high degree of sequence similarity between the *de novo* assembly and the reference genome, aligning them based on either consecutive *k*-mers or non-consecutive *k*-mers, i.e., with a fixed-size gap between any two *k*-mer matches on *both* the query and the reference sequences, can yield similar mappings. Figure 1 illustrates this idea. Here *k* = 2 and the gap size is 5 bp. The three cases in the figure demonstrate how non-consecutive *k*-mer matches can correctly map the query to the reference sequence in the cases of a perfect match, a deletion, and an insertion. Notice how three different subsets of *k*-mers function as alignment anchors. Gapped seeds can vastly reduce the search time and space, however spurious matches can arise due to the low sensitivity. We combine the use of non-consecutive *k*-mers with a false alignment pruning step in order to quickly and correctly identify candidate homologous regions of the query sequences.

For each chromosome 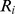, the hash table 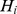 is loaded into the memory such that only the *k*-mer positions contributing to some selected subsets are filtered in. Let 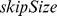 denote the number of bases to be skipped between any two consecutive *k*-mers in a particular set such that 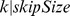 and 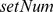 be the total number of *k*-mer sets to be loaded such that 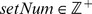 and 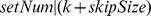. Let 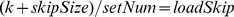. For each 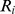, only the positions divisible by 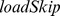 from 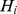 are loaded. This guarantees that 1) strictly 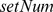 subsets of *k*-mers, with 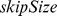 bases between any two consecutive *k*-mers in a particular set, are loaded into the memory, and 2) the first *k*-mer in any two consecutive sets are exactly 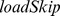 bases apart. For example, let *k* = 10, 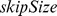 = 80, and 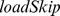 = 45, 2 sets of *k*-mers starting at these positions will be loaded, 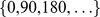 and 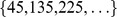. Using multiple *k*-mer subsets ensures that the algorithm caters to all three cases above. Then the program scans each query 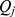 from left to right in both orientations and attempts to align it to *k* by matching as many *k*-mers between them as possible. 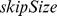 bases are skipped on both 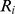 and 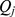 after every match. Only maximal unique alignments, i.e., stretches of *k*-mer matches that occur once and cannot be extended further in either direction, longer than a minimum user-defined threshold (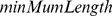) are retained. A maximal unique aligned region is created by intersecting the position lists between the current aligned region and the next *k*-mer until the intersection contains a unique location. Nearly 80% of the human genome is composed of unique 25-mers [8], thus each maximal unique aligned region is expected to emerge after a few intersections. Once no more *k*-mer matches can be found for the current alignment, the process restarts at the base following the last aligned region until the end of 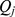 is reached. The search is applied independently in both orientations of 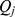. In a single orientation, each base is scanned exactly once.

**Figure 2:**
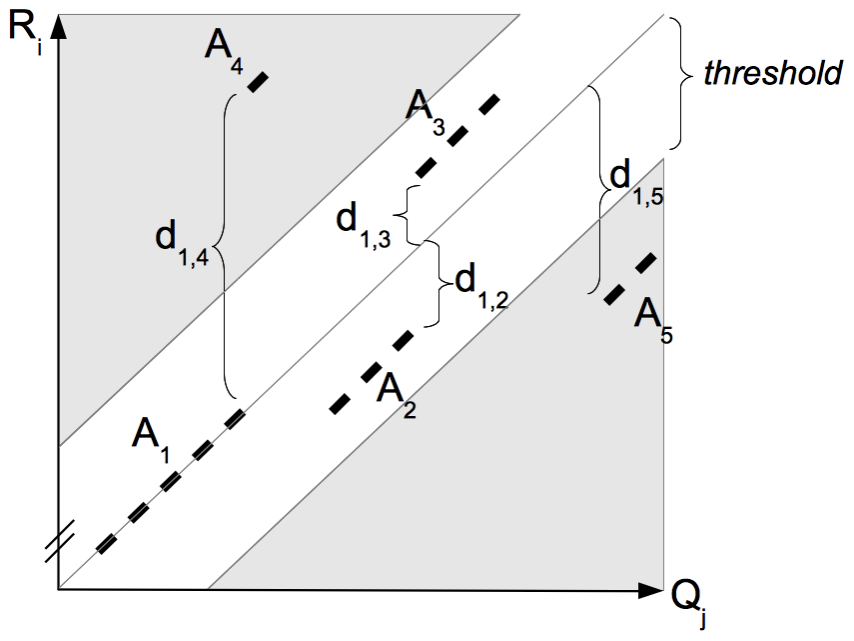
Removal of spurious matches between 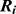 and 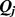. The initial alignment consists of five aligned regions 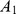 to 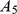. 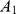 is the longest region and serves as an anchor for a true alignment. The distances 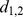 to 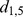 measure the distance differences on 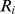 of 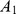 to 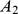 and 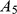, respectively. 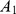, 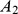, and 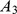 are kept as candidates for the true alignment because 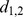 and 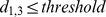.

Figure 2 demonstrates how NSIT removes spurious matches. Define the distance between any two aligned regions to be the difference between their 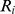 coordinates. The longest aligned region serves as an anchor for a true alignment and only alignments within 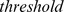 distance from it are retained as parts of a true alignment candidate. Since most structural variations in the human genome range from about 1 kb to a few Mb in size, we randomly tested different values for 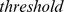 in the range of 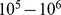. We found no change in the results and arbitrarily set 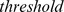 to 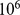. The alignment score is defined as the total number of bases in the alignment. Each query maintains 48 scores, one for each reference chromosome in a specific orientation. Next the algorithm assigns the most probable target chromosome(s) and orientation(s) to each query. Let 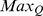 be the total number of *de novo* sequences and let 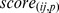 be the alignment score between 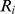 and 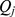 in the orientation 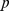 where 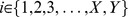, 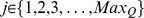, and 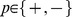. Since a spurious alignment may occur randomly, we expect the distribution of the scores from chance matches of any given 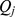 to follow the Gaussian distribution, while the true alignment score of 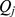 should deviate significantly from them. Let 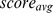 and 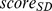 be the mean and standard deviation of all scores 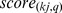 such that 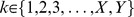, 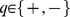, and 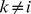 when 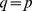. The algorithm assigns any chromosome 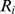 in orientation 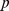 to 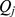 if and only if 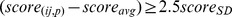, which means 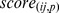 has p-value 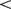 0.01, signaling a possible real alignment.

The high similarity between the query and reference sequences allows the majority of scaffolds and contigs to be efficiently and correctly assigned in this manner. Nevertheless, the heuristic nature of the algorithm may render some queries assigned to multiple targets, incorrectly assigned, or non-assignable, which are discussed next. The time complexity of this phase is linear to the human genome size and the space requirement is bounded by the size of the largest reference chromosome, i.e., chromosome 1, plus the size of the largest query, which in our experiments required <1 GB in a 32-bit environment.

### Query Alignment

Based on the assignments, in this phase the algorithm aligns the following 4 groups of *de novo* sequences to the reference genome in the manner similar to the previous phase. The main differences are that all of the indexed *k*-mers are used in order to increase the alignment sensitivity and no skipping is performed.

The first group contains the contigs and scaffolds that are assigned at least one target reference chromosome and alignment orientation. These sequences are aligned to the reference chromosomes according to their assignments. The second group contains the sequences unassigned. Any sequences not aligned to their assigned chromosomes for more than half their lengths are considered incorrectly assigned, constituting the third group. Lastly, since the human genome contains much repetitive content, it is possible that the *de novo* assembler may fuse regions from different chromosomes into a single scaffold. As a result, some of these regions would not align to the assigned chromosomes and appear as possible novel sequence candidates, even though they are not novel. Such false positive novel sequence candidates can be detected as large unaligned regions in the alignment results of the first three groups. Based on our input *de novo* assemblies, these regions are usually larger than 2.5 kb, therefore we set our cutoff to that value. The last three groups of *de novo* sequences are aligned to *all* reference chromosomes in *both* orientations.

The majority of contigs and scaffolds are assigned accurately and uniquely (See Results and Discussion). Thus, this phase approximately runs in linear time in practice. Resulting alignments are extended by direct base comparison as far as possible in both directions. Alignment overlaps are resolved by giving a higher priority to the longer alignment. A tie is broken at random. For any query, the alignment with the highest score is chosen as the true alignment. The final unaligned regions in the *de novo* queries are passed on as novel sequence candidates to BLASTn, which performs fine-scale sequence alignments between these candidates and the reference human genome. Note that only the reference chromosomes are included in NSIT steps, but the non-chromosomal reference sequences, e.g., unlocalized and unplaced scaffolds, are also included in the post-processing steps with BLASTn. (Our analysis excludes all alternate loci scaffolds.) A human reference assembly typically contains a small number of non-chromosomal bases. For example, around 22 Mb, 6 Mb, and 11 Mb are present in the NCBI36, GRCh37, and GRCh38 assemblies, respectively. The initial exclusion of these sequences may increase the number of novel base candidates to be filtered with BLASTn, but since their collective sizes are much smaller than the rest of the genome, we expect them to minimally impact the total run time. The regions unaligned by BLASTn are searched for known repeats via RepeatMasker. Final unaligned regions that are 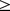
100 bp long and not known repeats are reported as novel sequences.

## Results and Discussion

We set out to identify the novel sequences in three *de novo* human genome assemblies: NA18507, YH, and NA12878 (Table 1). Our goal was to evaluate NSIT's performance, both in terms of the result correctness and the program's efficiency. The first two input genomes were assembled with SOAPdenovo and their novel sequences were reported in [1]. We compared our results to such novel sequences in this paper. NA12878 was assembled with ALLPATHS-LG [12]. To our knowledge, its novel sequences have not been described via this algorithmic route elsewhere. Among the three assemblies, NA12878 was the most recent one and contains the fewest number of scaffolds and no contigs. In contrast, NA18507 was sequenced with the earliest technology and is more fragmented. We performed our experiments on a 32-bit Linux machine with Intel Xeon 3.00 GHz quad-core processors, 4GB of RAM, and 500 GB of hard disk. The amount of RAM used across all experiments was <2 GB.

**Table 1:** Summary of input *de novo* whole human genome assemblies.

	NA18507	YH	NA12878
Sequencer	Illumina GA1	Illumina GAII	Illumina GAII and HiSeq
Average read lengths (bp)	35 bp	55 bp	76 bp and 101 bp
Average insert sizes (bp)	2 kb	9.6 kb	155 bp, 2.5 kb, and 35.3 kb
*De novo* assembler	SOAPdenovo	SOAPdenovo	ALLPATHS-LG
Data availability date	December 2009	December 2009	January 2011
Number of contigs	144,443	136,926	0
Min contig length (bp)	100	100	N/A
N50 contig length (bp)	266	211	N/A
Max contig length (kb)	6.8	4.5	N/A
Total contig length (Mb)	32.5	27.7	N/A
Number of scaffolds	170,434	48,160	3,331
Min scaffold length (bp)	102	108	1,471
N50 scaffold length (Mb)	0.1	0.5	12.1
Max scaffold length (Mb)	0.8	3.5	48.7
Total scaffold length (Gb)	2.7	2.9	2.8
Total assembly length (Gb)	2.7	2.9	2.8

### Chromosome Assignment and Query Alignment

**Figure 3:**
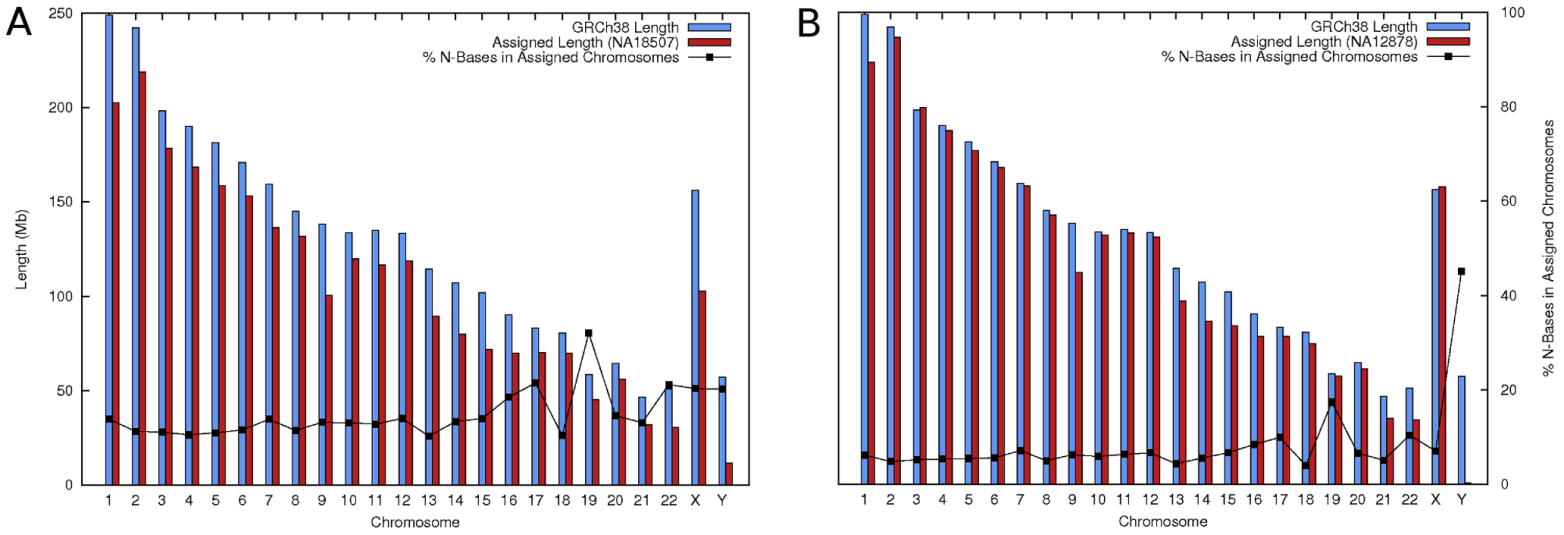
Results of the Assignment Phase. For all three input assemblies, the total numbers of bases assigned per chromosome closely mirrored the reference chromosome lengths. (YH result is shown in Figure S1.) The numbers of ambiguous bases (N bases) in the *de novo* sequences assigned were consistent with the actual chromosomal repeat contents, i.e., Chr 17, 19, 22, X, and Y are known to be repeat-rich. a) NA18507 assembly has a smaller N50 size, which made it more difficult to map yielding fewer bases assigned per chromosome. b) The graph shows that Chr Y was assigned only few bases, which concurs with the fact that NA12878 was a female donor.

Results in Table 2 show that NSIT was able to assign a chromosome and an orientation to 94–99% of the *de novo* sequences across all three input assemblies. Furthermore, 93–96% of these were assigned *uniquely* to a chromosome and an orientation. Figure 3 and Figure S1 show that the number of bases assigned per chromosome consistently reflected the true chromosome lengths. In addition, the *de novo* sequences and their assigned reference regions exhibited very high sequence identities of 90–97%. The line graphs in the figures display the proportions of N bases present in the assigned *de novo* sequences per chromosome. Stretches of ambiguous or N bases in a *de novo* assembly indicate sequence gaps, which are usually induced by unresolved repeats. The N-base proportions in our results concurred with the exceptionally high repeat contents of chromosomes 17, 19, 22, X, and Y [13]–[17], and thus are highly indicative of correct assignments.

**Table 2:** Result statistics of the Chromosome Assignment and Query Alignment Phases.

	NA18507	YH	NA12878
**Parameters**			
*k*	12	12	12
Minimum MUM length	150	200	200
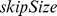	60	84	84
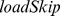	4	6	6
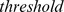	10^6^	10^6^	10^6^
**Assigned Sequences**			
Total bases assigned (%)	93.6	96.2	99.4
Unique bases assigned (%)	92.8	95.0	96.3
Sequence identity to reference (%)	97.0	96.6	90.3
Chromosomes with %N-bases > 1 or 2 SD	17, 22, X, Y/19	19/X, Y	19/Y
**Unassigned Sequences**			
Total (Mb)	171.8	108.0	17.4
Median/Mean/N50 sizes (kb)	0.3/0.9/4.5	0.2/0.8/6.6	1.2/3.4/7.2
**Incorrectly Assigned Sequences**			
Total bases (Mb)	7.7	3.4	0.7
Median/Mean/N50 (kb)	0.3/0.7/2.2	0.2/0.8/6.0	1.2/3.6/6.3
**False Positive ** ***de novo*** ** Sequences**			
Total bases (Mb)	4.0	10.4	15.6
Median/Mean/N50 sizes (kb)	3.3/3.9/3.8	3.5/4.4/4.4	3.6/4.4/4.5
**Total run time (min)**	122	93	97

Next the algorithm aligned the assigned sequences, the unassigned sequences, the incorrectly assigned sequences, and the false positive novel sequences to the reference genome. In all three assemblies, ≥94% of the bases were in the first group (assigned) and the other three groups contained sequences that were only a few kb short or less, and highly enriched in N bases. The total run times of NSIT against the GRCh38 reference assembly were 122 min, 93 min, and 97 min for NA18507, YH, and NA12878, respectively. NA18507 is the most fragmented assembly among the three, thus it was more difficult to map and required a longer run time. The NA12878 assembly has a very large N50 size of 12.1 Mb, in comparison to the other two (Table 1). This could result in a higher run time (120 min in our experiment) due to the larger sizes of incorrectly assigned and false positive *de novo* sequences. We solved this problem by breaking its *de novo* sequences to approximate the size of YH *de novo* sequences, and used this modified assembly as the input. With this fix, we achieved similar run times of 93 and 97 min for YH and NA12878, without affecting the NA12878 novel sequence integrity. For input assemblies with a large N50 value, we recommend applying this step to speed up the run time. In general, *de novo* genome assemblies should comprise larger scaffolds as technology evolves, and this simple solution can be adopted for future input assemblies.

### Novel Sequences

Based on the alignments obtained, the unaligned regions became our novel sequence candidates and we post-processed them with RepeatMasker and BLASTn. RepeatMasker filtered out known repeats and BLASTn served two purposes: to remove the sequences which aligned to the reference sequence but were missed by NSIT and to screen for possible sequence contaminants. A similar approach was taken in [1], where the authors used BLAT and LASTZ aligners to narrow down the candidates and BLASTn to finalize the list of novel sequences for NA18507 and YH.

We first assessed our result correctness by comparing NSIT's novel sequences to those reported in [1], in which the authors compared the NA18507 and YH *de novo* assemblies to the NCBI36 reference assembly and identified 4.8 Mb and 5.1 Mb of novel sequences, respectively. To mirror [1], we used NSIT to align the two assemblies to NCBI36 and located 50.3 Mb and 62.0 Mb of novel sequence candidates, respectively. We aligned the candidates to NCBI36 with BLASTn. The unaligned regions were treated as novel sequences and were compared to those identified in [1]. We found that more than 99% the novel sequences reported in [1] were included in our initial candidate novel sequence sets for both NA18507 and YH. When filtering with BLASTn, depending on its threshold stringency, our approach narrowed down to 5.1–6.2 Mb of novel sequences in both *de novo* assemblies and 92.4–99.1% of the novel bases in [1] were present within our novel sequences. This validated our results and also showed that our method is highly accurate. We speculate that the differences seen in the final sets of novel sequences were likely due to the details of BLASTn, and possibly BLAT and LASTZ, parameter settings.

Next we aimed to determine the most up-to-date novel sequences for all three *de novo* assemblies. First we used NSIT to align the assemblies to the latest human reference assembly, GRCh38, and located 46.0 Mb, 57.4 Mb, and 65.4 Mb of novel sequence candidates in NA18507, YH, and NA12878, respectively. We applied similar BLASTn thresholds to further align the candidate sequences to GRCh38, which reduced their sizes to 2.2 Mb, 2.4 Mb, and 3.8 Mb, respectively. Known repeats were filtered via RepeatMasker, yielding 1.2 Mb, 1.2 Mb, and 1.3 Mb of candidates. Lastly, we detected 130.0 kb of Epstein-Barr virus sequence contaminants in NA18507 and 287.4 kb of zebrafish (Danio rerio) repeat sequence contaminants in NA12878. (Details are discussed below.) As a result, the final identified novel sequences are 1,059,744 bp, 1,242,987 bp, and 1,040,732 bp for NA18507, YH, and NA12878, respectively. The updated novel sequence sizes are substantially smaller than the previously reported numbers in [1] most likely because the GRCh38 assembly is more complete than the NCBI36 assembly and we removed known repeats. Our results also imply that the human pan-genome size may not be as large as previously predicted. Overall, the post-processing steps required 74 min, 100 min, and 182 min, respectively, where 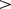
50% of the time on average was for running RepeatMasker on its server. Table 3 summarizes our findings.

**Table 3:** Novel sequences of NA18507, YH, and NA12878 *de novo* whole genome assemblies.

	NA18507	YH	NA12878
Candidate bases from NSIT (Mb)	46.0	57.4	65.4
Remaining bases after *post*-processing (Mb)	1.1	1.2	1.0
Total *post*-processing time (min)	74	100	182
– BLASTn time (min)	17	40	105
– RepeatMasker time (min)	57	60	77
Novel bases aligned with:			
– NA18507 Novel bases (Mb)	-	0.7	0.7
– YH Novel bases (Mb)	0.7	-	0.7
– NA12878 Novel bases (Mb)	0.7	0.7	-
– Human Decoy Sequences (Mb)	0.7	0.8	0.8
– HuRef Assembly (Mb)	0.6	0.6	0.6
– CHM1 Assembly (Mb)	0.4	0.4	0.4
– Chimpanzee Genome (Mb)	0.7	0.7	0.7
– Gorilla Genome (Mb)	0.7	0.7	0.7
Possible sequence contamination:			
– EBV Genome (kb)	130.0	0	0
– Mouse Genome (kb)	0.4	7.0	0.7
– Zebrafish Repeats (kb)	44.5	63.8	287.4

**Figure 4:**
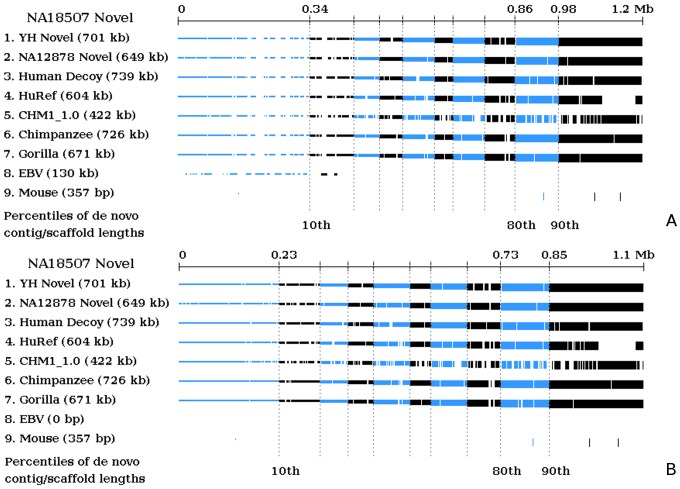
Comparing NA18507 novel sequences with other sequences. We sorted the novel sequences of NA18507 from left to right ascendingly by the size of their originating contigs or scaffolds. The result shows high overlaps between the three sets of novel sequences. In addition, around 0.4–0.7 Mb of them are present in the human decoy sequences, HuRef assembly, CHM1 Assembly, Chimpanzee genome, and Gorilla genome. In addition to illustrating the sequence comparisons, NSIT graphical viewer can aid in removal of large sequence contamination. a) It is clear from the figure that certain novel sequence regions toward the left (all from contigs) did not align to any of the above sequences. These regions amounted to approximately 130 kb and instead aligned with high confidence to the Epstein-Barr virus (EBV) genome, suggesting possible sequence contamination. b) After removal of such regions, 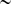
1.1 Mb of novel sequences remained and the overlaps with the other sequences were unchanged.

We characterized the identified novel sequences using BLASTn. When compared among the three sets, about 0.7 Mb of the novel bases overlapped. Across all three sets, 0.7–0.8 Mb aligned to the human decoy sequences [18], 0.6 Mb aligned to the HuRef assembly, and 0.4 Mb aligned to the CHM1 assembly, respectively. Moreover, around 0.7 Mb of each set aligned to the Chimpanzee and Gorilla genomes. (Table 3) Such extent of sequence overlap supported that these were valid DNA sequences and the non-overlapping portions were likely population- or individual- specific, as described in [1]. To further illustrate, we used NSIT graphical viewer to depict the comparisons shown in Figures 4, S2, and S3. Here the novel sequences were arranged in the ascending order of the lengths of their originating *de novo* contigs or scaffolds, i.e., the novel sequences originating from contigs would be on the left whereas the ones originating from scaffolds would be on the right. The dashed vertical lines represent the percentiles of such length. Mapped novel sequence regions are indicated by the colored bars. The bar height indicates the percentile range. The graphs show that the mappings occurred almost uniformly across all novel sequences. This is the first study which illustrates graphically how the novel sequences are aligned to other selected sequences. In previous studies, pie charts or Venn diagrams were used to show the sizes of sequence overlaps. A side benefit of this graphical representation is that it allows for quick high-level identification of possible sequence contaminations in the novel sequences. For example, Figure 4A shows a region toward the lower percentile of the novel sequences which clearly does not map to any of the aforementioned sequences. A closer look revealed that this lower-end contig-rich area contained as high as 130.0 kb of the NA18507 novel sequences that did not match with any existing human genome assemblies, or any other closely related species, but aligned with extremely high confidence and near perfect sequence identity to the Epstein-Barr virus genome. We removed these sequences from our result (Figure 4B). Note that these 130.0 kb were included as a part of the NA18507 novel sequences and *de novo* assembly reported in [1]. Another example is illustrated in Figure S3. Initially, we found 1.3 Mb of novel sequence candidates for NA12878, which was noticeably larger than the other two sets. Figure S3A shows several large unaligned regions in this candidate set, primarily from the shorter *de novo* scaffolds. We further screened the candidates with known repeats from other organisms and removed 287.4 kb of zebrafish-specific repeats from it, in comparison to the 44.5 and 63.8 kb found in NA18507 and YH novel sequences, respectively. The updated graph has similar characteristics to the previous two sets and is shown in Figure S3B.

### Parameter Settings in NSIT

NSIT has four user-defined parameters dictating the program's efficiency: 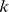, 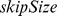, 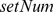, and 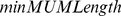. For best performances, we recommend the following settings. 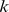 is usually set to 8–16, where a multiple of 3 (codon size) is preferred. NSIT requires that the following conditions hold: 1) 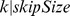, 2) 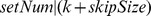, and 3) 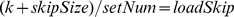. We empirically determined over a range of experiments that a value of 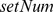 between 10–20 sets yields the best run times and that at least three 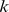-mers should match in order to form an assignment. Since nearly 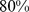 of the human genome is composed of unique 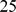-mers [8], 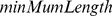 should be larger than 25 bp. The 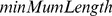 threshold should also be small enough to cater to the chromosome assignments for smaller contigs, which can be determined based on the input assembly's sequence size distribution.

Let's take the YH *de novo* assembly as an example. 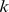 was set to 12. Only about 0.5% of the assembly are contigs of size shorter than 200 bp and we set 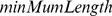 to this value. To form an assignment with at least three 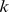-mers, this equation must hold: 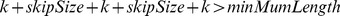 or 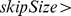 82. We thus set 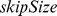 to 84 so that it was also divisible by 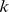. Since 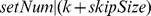 and 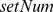 should be between 10–20, we selected 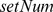 to 16, yielding 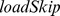 of 6. We set the parameter values in this manner in all our experiments.

Parameter adjustments are possible. Decreasing 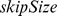 and 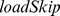 (increasing 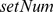) causes more 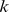-mers to be loaded during the 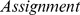 phase, thus increasing sensitivity and the phase's run time. As a result, fewer bases remain to be aligned against the entire reference genome in the 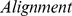 phase, thus decreasing the phase's run time. A small 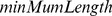 increases the program's sensitivity but also the number of possible spurious matches, and vice versa. Our results show that NSIT is robust in terms of handling changes in parameter values outside the recommended ranges (Figures S4 and S5). The run times and novel sequence candidate sizes did not vary significantly. It was not possible to compare different values of 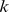 while keeping other parameters constant. In general, a smaller 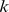 increases sensitivity but also the number of spurious matches, and thus the run time, and vice versa.

### Performance Comparison with Existing Methods

We compared NSIT with BLAT, LASTZ, and QueryLookupTable. A pipeline of BLAT and LASTZ was adopted in [1] to identify the novel sequences of NA18507 and YH. QueryLookupTable was used for mapping the contigs of NA12878 to the reference sequence in order to construct the final *de novo* assembly in [12] and it requires a 64-bit machine. Although it was not used for the task of discovering the novel sequences, the contig fragments unaligned by QueryLookupTable could serve as candidates. All experiments here were run on a 64-bit Linux server with Intel Xeon 2.67GHz multi-core processors, 16GB of RAM, and 600GB of hard disk.

To compare NSIT with BLAT and LASTZ, we aligned the YH *de novo* assembly to the NCBI36 by following the steps and parameter settings in [1]. The seed size was set to 12 for both programs to keep the value of 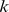 consistent. BLAT was run first with the -fastmap and -ooc options enabled for speed and -stepSize = 84 to reflect our 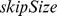. The memory usage was 440 MB and the time taken was 127 min. Next we refined the alignments using LASTZ with step size = 90, which took 75 min and 320 MB of RAM. (We also tested with step sizes of 10, 20, and 48. The results were very close to one another but the run times increased substantially.) The number of novel base candidates found was 35.8 Mb and the total run time was 202 min, prior to post-processing. In contrast, the combination of NSIT and the post-processing steps required 126 min and 2GB of RAM, yielding 1.1 Mb of novel sequences. For QueryLookupTable, we followed the parameter settings used in [12]. The program required 28.3 hrs and 7GB of RAM to align the *de novo* NA12878 contigs to the GRCh38 reference chromosome 1 alone, thus we did not proceed with the rest of the genome. Here we ran NSIT on NA12878 contigs (not the scaffolds) against the entire reference assembly, using 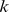 = 12, 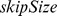 = 60, 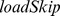 = 4, and 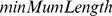 = 150, and it required 114 min and 2GB of RAM. About 71 Mb of novel sequence candidates were discovered.

These results show that NSIT was faster than the other programs by large margins. The difference was likely due to the fact that NSIT caters specifically to the problem of novel sequence identification, while the others are more general aligners and thus include extra steps unnecessary for this problem. For example, NSIT skips the bases both on the reference and query sequences in the *Assignment Phase* while LASTZ has an option of skipping the bases on the reference string only. Although NSIT requires more memory, 2GB is commonly available in today's computers.

## Conclusion and Future Directions

In this paper we describe NSIT, an algorithm for identifying novel sequences in a *de novo* human genome assembly. Our program includes a novel sequence graphical viewer, requires modest compute resources, is accurate, and is several times faster than other programs previously applied to this task. It runs in both 32-bit and 64-bit Linux operating systems. Results show that NSIT is robust and applicable to *de novo* assemblies from various NGS platforms and assemblers; it was tested with three *de novo* assemblies with satisfactory results. We therefore conclude that NSIT is a highly suitable software for this task and can be very useful as more *de novo* whole human genome assemblies become available. NSIT is open source and available under the GNU General Public License (GPLv3) at www.sourceforge.net/projects/nsit.

Since we successfully used NSIT to align the NA12878 contigs to the reference sequence above, we plan to explore its related applications, such as whole genome scaffolding and decoy sequence search. The human decoy sequences [18] are human DNA sequences that are missing from the GRCh37 reference assembly. When included in a SNP calling pipeline, they can help reduce the error rates substantially. The decoy sequences were identified by aligning HuRef contigs, NA12878 contigs, GRCh37.p4 patches, and human BAC/fosmid clones from GenBank to GRCh37 with BWA-SW [19], and extracting the unmapped regions. Their total size is 35.4 Mb where 73% are known repeats. To investigate NSIT's applicability, we aligned the NA12878 contigs to the reference chromosome 1 with BWA-MEM [20], which is recommended over BWA-SW for long sequences. k was set to 12. The memory usage was 525 MB and time taken was 24.5 hrs on our 64-bit server. Next we extracted only high-confident alignments and found that they highly overlapped with NSIT's chromosome 1 result. In addition, approximately 4.0 Mb of the decoy sequences originated from NA12878 and around nearly 90% of those were present in NSIT's NA12878 novel sequence candidates. BWA-MEM is a much more sensitive and accurate aligner than NSIT, however NSIT's speed and high degree of result overlap suggest that it could be used as a filtering step to reduce the search time and space for future sets of decoy sequences. Our results warrant further investigation as more *de novo* human genome assemblies, especially ones from different populations, become available.

## Supporting Information

Figure S1 **Results of the Assignment Phase for YH.** Similar to NA18507 and NA12878 results, the total lengths of YH *de novo* sequences assigned per chromosome closely mirrored the reference chromosome lengths. The amounts of N bases assigned per chromosome were also consistent with actual chromosomal repeat contents.
(TIFF)

Figure S2
**Comparing YH novel sequences with other sequences.** We performed a similar analysis to Figure 4 here and found that the sequences overlapped similarly. The graph suggests no obvious contamination traces, however we found that 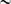
7 kb of YH novel sequences aligned with high confidence to the mouse genome, in comparison to 0.4 and 0.7 kb in the other two sets of novel sequences.(TIFF)

Figure S3 **Comparison of NA12878 novel sequences with other sequences.** a) The initial post-processing steps yielded 1.3 Mb of novel sequence candidates for NA12878, which was noticeably larger than the other two sets of novel sequences. We plotted it against other sequences and found several sizable unaligned regions, especially in candidates originating from shorter scaffolds (toward the left). This again suggested possible sequence contamination. b) Further screening with RepeatMasker detected 287.4 kb of zebrafish repeats in the candidates. Removing such repeats resulted in 1.0 Mb of novel sequences identified for NA12878.
(TIFF)

Figure S4
**Effect of varying NSIT's parameter values on the run time.** a) Varying 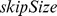, *k* = 12, 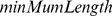 = 200, and 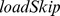 = 6 b) Varying 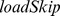, *k* = 12, 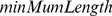 = 200, and 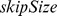 = 84 c) Varying 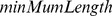, *k* = 12, 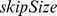 = 84, 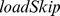 = 6. The run times did not vary significantly.(TIFF)

Figure S5
**Effect of varying NSIT's parameter values on the novel sequence candidate size.** a) Varying 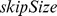, *k* = 12, 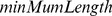 = 200, and 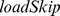 = 6 b) Varying 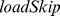, *k* = 12, 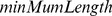 = 200, and 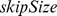 = 84 c) Varying 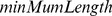, *k* = 12, 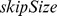 = 84, 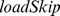 = 6. The novel sequence candidate sizes did not vary significantly.(TIFF)
