# Supplementary figures and images for: NSIT: Novel Sequence Identification Tool
Source: PLoS One. 2014 Sep 29;9(9):e108011. doi: 10.1371/journal.pone.0108011 (PMC4180056; doi:10.1371/journal.pone.0108011)

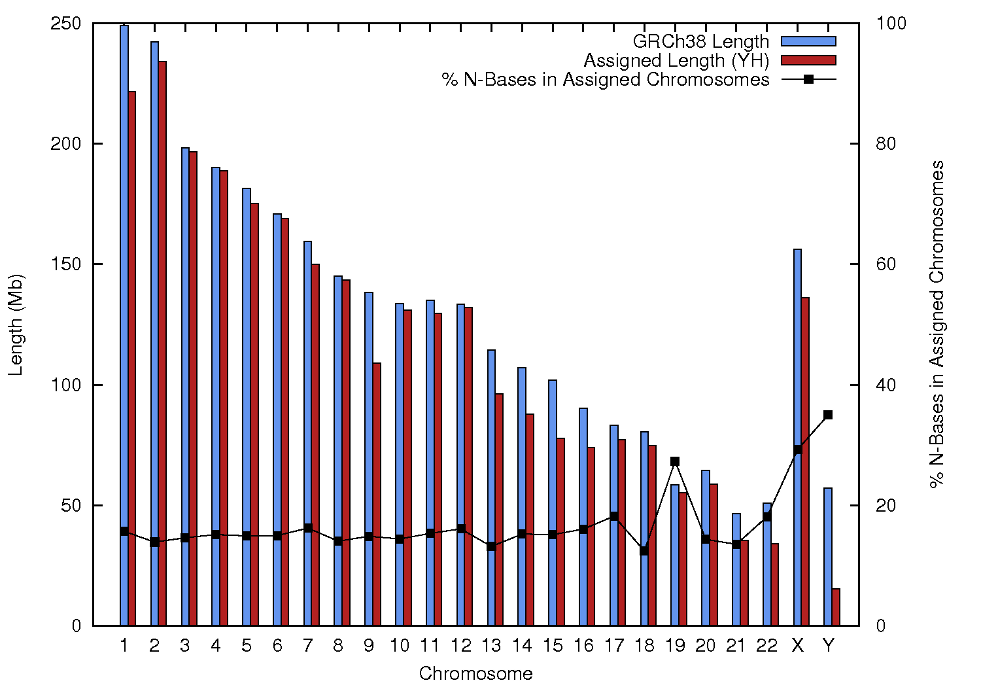

Supplement: Figure S1 — Results of the Assignment Phase for YH. Similar to NA18507 and NA12878 results, the total lengths of YH de novo sequences assigned per chromosome closely mirrored the reference chromosome lengths. The amounts of N bases assigned per chromosome were also consistent with actual chromosomal repeat contents. (TIFF) [file pone.0108011.s001.tiff]

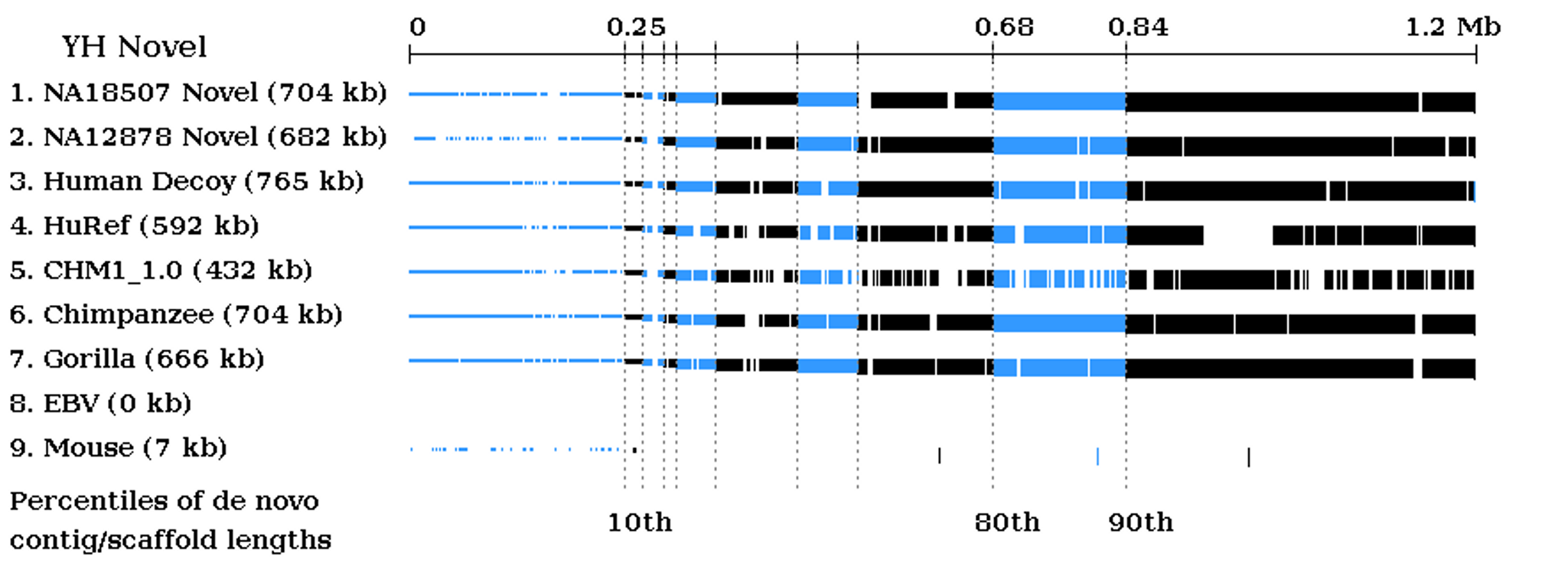

Supplement: Figure S2 — Comparing YH novel sequences with other sequences. We performed a similar analysis to Figure 4 here and found that the sequences overlapped similarly. The graph suggests no obvious contamination traces, however we found that 7 kb of YH novel sequences aligned with high confidence to the mouse genome, in comparison to 0.4 and 0.7 kb in the other two sets of novel sequences. (TIFF) [file pone.0108011.s002.tiff]

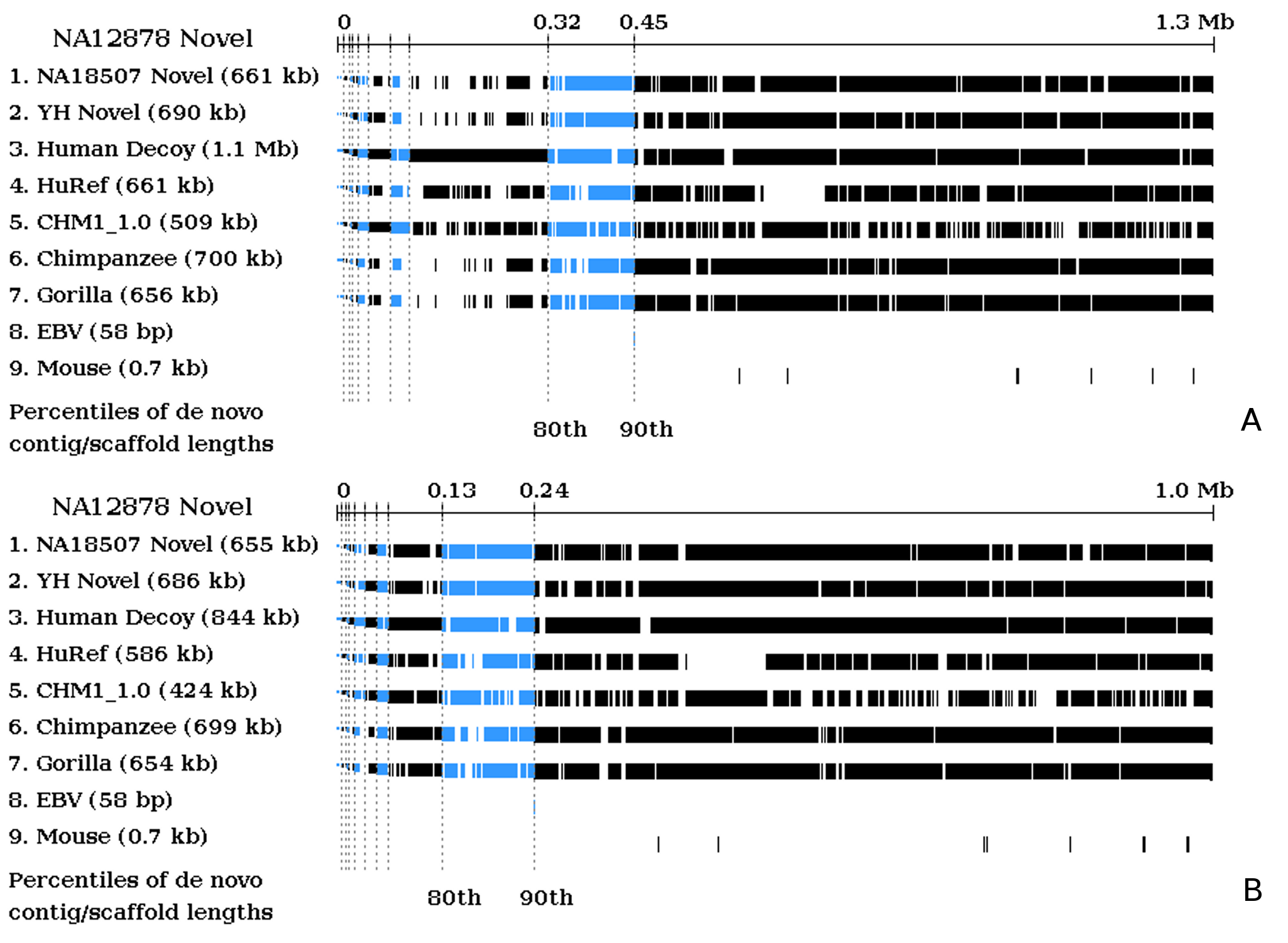

Supplement: Figure S3 — Comparison of NA12878 novel sequences with other sequences. a) The initial post-processing steps yielded 1.3 Mb of novel sequence candidates for NA12878, which was noticeably larger than the other two sets of novel sequences. We plotted it against other sequences and found several sizable unaligned regions, especially in candidates originating from shorter scaffolds (toward the left). This again suggested possible sequence contamination. b) Further screening with RepeatMasker detected 287.4 kb of zebrafish repeats in the candidates. Removing such repeats resulted in 1.0 Mb of novel sequences identified for NA12878. (TIFF) [file pone.0108011.s003.tiff]

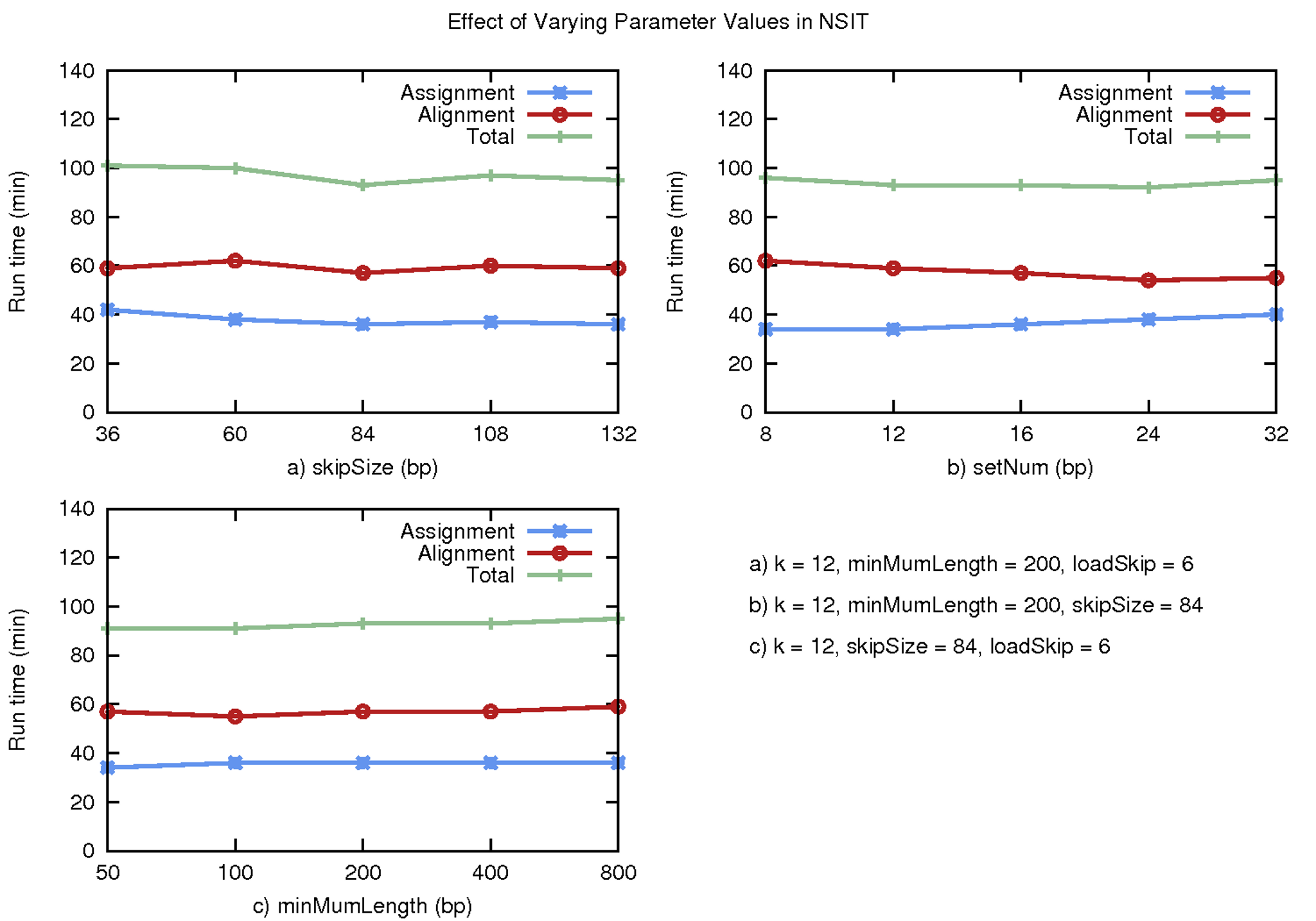

Supplement: Figure S4 — Effect of varying NSIT's parameter values on the run time. a) Varying , k = 12, = 200, and = 6 b) Varying , k = 12, = 200, and = 84 c) Varying , k = 12, = 84, = 6. The run times did not vary significantly. (TIFF) [file pone.0108011.s004.tiff]

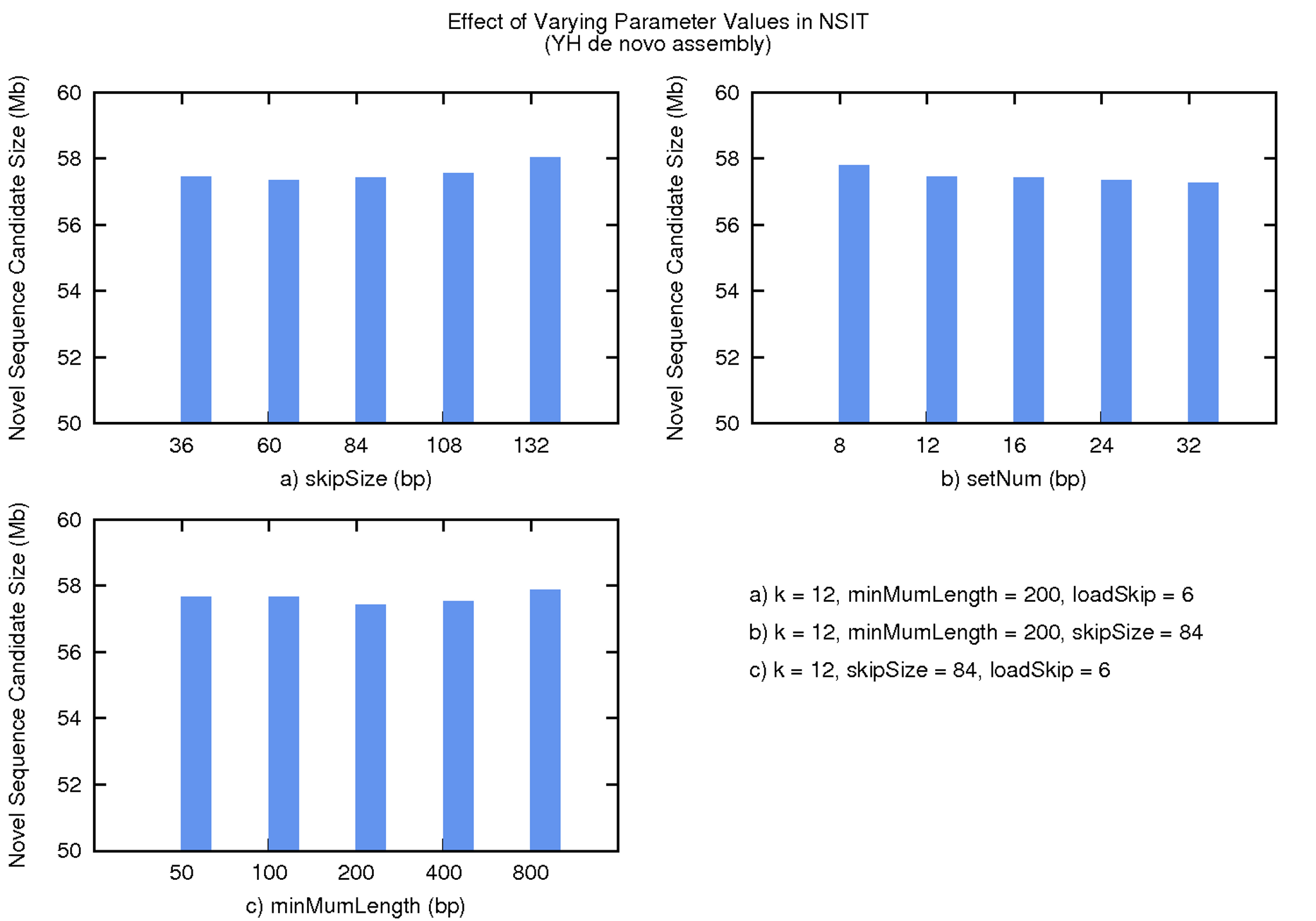

Supplement: Figure S5 — Effect of varying NSIT's parameter values on the novel sequence candidate size. a) Varying , k = 12, = 200, and = 6 b) Varying , k = 12, = 200, and = 84 c) Varying , k = 12, = 84, = 6. The novel sequence candidate sizes did not vary significantly. (TIFF) [file pone.0108011.s005.tiff]
